# The association between the food environment and adherence to healthy diet quality: the Maastricht Study

**DOI:** 10.1017/S1368980023001180

**Published:** 2023-09

**Authors:** Jeffrey Alexander Chan, Annemarie Koster, Simone JPM Eussen, Maria Gabriela M Pinho, Jeroen Lakerveld, Coen DA Stehouwer, Pieter C Dagnelie, Carla J van der Kallen, Marleen MJ van Greevenbroek, Anke Wesselius, Hans Bosma

**Affiliations:** 1 Care and Public Health Research Institute (CAPHRI), Maastricht University, Maastricht, The Netherlands; 2 Department of Social Medicine, Maastricht University, Maastricht, The Netherlands; 3 Department of Physical Medicine and Rehabilitation, Northern California VA Healthcare System, Martinez, CA, USA; 4 Department of Epidemiology, Maastricht University, Maastricht, The Netherlands; 5 Cardiovascular Research Institute Maastricht (CARIM), Maastricht University, Maastricht, The Netherlands; 6 Department of Epidemiology and Data Science, Amsterdam UMC, Vrije Universiteit Amsterdam, Amsterdam, The Netherlands; 7 Department of Internal Medicine, Maastricht University, Maastricht, The Netherlands; 8 School for Nutrition and Translational Research in Metabolism, Maastricht University, Maastricht, The Netherlands

**Keywords:** Food environment, Nutrition, Spatial epidemiology, Socio-economic status, Health inequality

## Abstract

**Objective:**

The purpose of this study is to determine if healthier neighbourhood food environments are associated with healthier diet quality.

**Design:**

This was a cross-sectional study using linear regression models to analyse data from the Maastricht Study. Diet quality was assessed using data collected with a FFQ to calculate the Dutch Healthy Diet (DHD). A buffer zone encompassing a 1000 m radius was created around each participant home address. The Food Environment Healthiness Index (FEHI) was calculated using a Kernel density analysis within the buffers of available food outlets. The association between the FEHI and the DHD score was analysed and adjusted for socio-economic variables.

**Setting:**

The region of Maastricht including the surrounding food retailers in the Netherlands.

**Participants:**

7367 subjects aged 40–75 years in the south of the Netherlands.

**Results:**

No relationship was identified between either the FEHI (B = 0·62; 95 % CI = –2·54, 3·78) or individual food outlets, such as fast food (B = –0·07; 95 % CI = –0·20, 0·07) and diet quality. Similar null findings using the FEHI were identified at the 500 m (B = 0·95; 95 % CI = –0·85, 2·75) and 1500 m (B = 1·57; 95 % CI = –3·30, 6·44) buffer. There was also no association between the food environment and individual items of the DHD including fruits, vegetables and sugar-sweetened beverages.

**Conclusion:**

The food environment in the Maastricht area appeared marginally unhealthy, but the differences in the food environment were not related to the quality of food that participants reported as intake.

As a result of increasing worldwide prevalence of obesity and diet-related chronic conditions (e.g. CVD), non-clinical interventional methods that may encourage an individual into healthier choices have gained notoriety^(1)^. In recent decades, the immediate surroundings of one’s home have been increasingly studied as a determinant to facilitate or become a deterrent to nutrient dense food intake^(2,3)^. Considerations in urban and suburban design are critical as distance travelled and surrounding options are components of food access. Coined the *Food Environment*, one aspect of this construct is that the proximity of food outlet (e.g. markets and restaurants) quantity and quality near one’s home may steer an individual’s dietary intake^(2,4)^.

Previous studies have shown associations of consuming higher amounts of fruits and vegetables in those living within proximity of supermarkets compared to living near a convenience store, a smaller market with limited healthy items typically at higher prices^(5–7)^. However, living nearby a higher density of fast-food restaurants was not found to equate to greater prevalence of a poorer diet in previous cross-sectional analyses^(2,8,9)^. An explanation of these inconsistent findings may be attributed to an array of factors such as location of sample collection, type of food retailers chosen and selected food groups as outcomes.

To date, most studies have been performed in the USA where there is generally greater contrast between neighbourhoods in terms of socio-economic and racial inequality compared to European and Asian societies^(10–13)^. Such geographic disparity and its possible reflection in the food environment can take on many forms and be distinct to a certain society. An example widely found in the USA are *food deserts*, areas where there is poor access to quality and affordable food, may not be as profound in other parts of the world^(14–16)^. It is therefore important to study the food environment across differing localities and cultures to determine if these findings remain.

Previous studies that focused on evaluating the food environment often relied on supermarkets and fast-food restaurants as sole indicators of individual food environments^(2)^. Relying on these specific food outlet types is limiting as it excludes many other varieties of stores and restaurants that individuals may regularly utilise. Gathering a collective of all the available food store types would be a comprehensive representation of what individuals may encounter in proximity to their home.

Finally, there is variability in outcome measures as most studies target fruit and vegetable intake while others focus on fast-food consumption, as well as specific nutrients. Making assumptions from one component of an individual’s diet is incomplete and may be misleading. Only few studies capture overall diet quality^(2,17)^, which may provide a better representation of individual’s nutrient consumption than by examining individual vegetable and fruit intake only.

In conjunction with the Geoscience and Health Cohort Consortium, data from the Maastricht Study were used to assess the association between the local food environment and dietary quality. Socio-economic indicators were evaluated to determine confounding on the latter association. We hypothesise that those living in the vicinity of higher quality food establishments (high inventory of fruits and vegetables, low processed foods, etc.) will have healthier dietary quality than those who do not. In addition, the associations between neighbourhood food environment and the consumption of sub-categories such as fruits, vegetables and sugar-sweetened beverages were examined consistent with neighbourhood food environment quality.

## Methods

### Study population

Data used were gathered from the Maastricht Study, an observational prospective population-based cohort study. The rationale and methodology have been described previously^(18)^. In brief, the study focuses on the aetiology, pathophysiology, complications and comorbidities of type 2 diabetes mellitus (T2DM) and is characterised by an extensive phenotyping approach.

Eligibility for participation was individuals between the ages of 40 and 75 years and living in the southern part of the Netherlands. Participants with and without diabetes were recruited through mass media campaigns, from the municipal registries and the regional Diabetes Patient Registry via mailings. Recruitment was stratified according to known T2DM status, with an oversampling of individuals with T2DM for reasons of efficiency. The present report includes cross-sectional data from the first 7689 participants, who completed the baseline survey between November 2010 and December 2017 with complete geographic data at the time of analysis. The examinations of each participant were performed within a time window of 3 months. Participants were excluded from analyses if their addresses could not be matched or had missing diet data (*n* 215), or missing data on education level (*n* 107) resulting in data from 7367 participants available for this study.

### Measures

A FFQ derived by van Dongen *et al*. that enabled the calculation of the Dutch Healthy Diet (DHD) index was completed by all participants at baseline entry of the study between 2010 and 2017^(19)^. A detailed description of the DHD can be found elsewhere^(20)^. In short, the DHD is a self-reported questionnaire with a scale of 0–150 that measures level of dietary adherence to the 2015 Dutch dietary guidelines with the overall highest score (150) corresponding to full compliance^(20)^. Fifteen food categories based on the Dutch dietary guidelines in 2015 comprise of the questionnaire which include healthy and less healthy components. The healthy components consist of vegetables, fruit, wholegrains, legumes, fish, dairy, nuts and tea. The less healthy components, with cut-offs for moderate consumption, include fats and oils, coffee, red meat, processed meat, sweetened beverages and fruit juice, alcohol and salt. Each food item was assigned a score of 0–10 with 10 being optimal. Data on filtered and unfiltered coffee were not included in the FFQ for the Maastricht Study as data collection began in 2010 and the DHD index was released in 2015. This component could not be derived as a result. The values for the DHD score for our study therefore ranged from 0 to 140.

### Food environment

Geo-data were collected as part of the Geoscience and Health Cohort Consortium (GECCO)^(21)^. GECCO data were linked to individual addresses from the Maastricht Study creating individual food environments for each of the participants. Data on the food environment in GECCO were based on the LOCATUS database consisting of a comprehensive audit taken every 2–3 years of available food outlets in the study region that included markets and restaurants^(22,23)^. GECCO-generated exposures to the food environment in 2015 or 2017 were matched to the year closest to their completion of the FFQ. The previous two LOCATUS audits before 2015 showed little change in food retailers compared to 2015 in the Maastricht region^(24,25)^. Thus, participants who enrolled in 2010–2015 and those who enrolled in 2016–2018 were combined in the 2015 and 2017 group, respectively.

**Figure 1:**
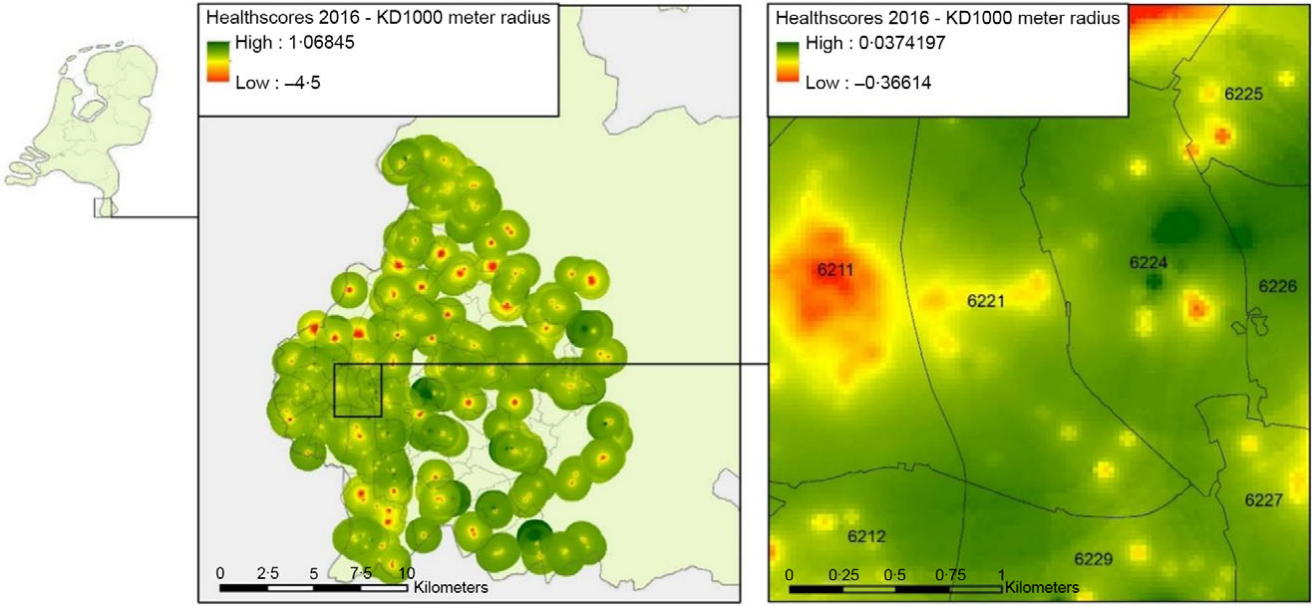
Heat map of food retailers by quality (FEHI) in Maastricht, NL. Green areas represent healthier food densities; red areas represent least healthy. FEHI, Food Environment Healthiness Index.

Using the types of food outlets from LOCATUS, a Delphi study conducted by an expert panel was performed to create an aggregate measure of the quality of the retail establishments in the Netherlands, generating the Food Environment Healthiness Index (FEHI)^(26)^. The FEHI index classifies each type of food retailer in relation to the nutritional quality of the foods they offer on a scale from –5·0 for the least healthy to +5·0 for the healthiest (see online supplementary material, Supplemental Table 1). Thus, a positive score is considered healthy and a negative score is less healthy. For example, a fish retailer and a supermarket were given a score of 2·8 and 1·8, respectively. A liquor store and confectionary shop were assigned values of –4·6 and –4·7. All scores of all food outlets averaged to zero in a kernel around the outlets, creating a heatmap with assigned FEHI scores for the full region (Fig. 1). An analysis was conducted evaluating a participant’s broader food environment using the FEHI that was assigned to their address area. In addition, the density of the six most common types of food outlets frequented was individually evaluated and included in the main analysis: local food shops (LF) including a butcher shop and bakery, fast food (FF), food delivery (FD), restaurants (RS), supermarkets (SM) and convenience stores (CS). The individual count of each of these establishments was calculated for each participant’s address. A Euclidian buffer zone of 1000 m was used as a reference distance from an individual address in relation to nearby food outlets. This allowed each participant to have their own residential food environment analysed. The distance of 1000 m was chosen in the main analysis as it is the best estimate within walking and cycling range for the average individual to travel to each food outlet, accounting for suburban neighbourhoods^(27,28)^.

### Covariates

Covariates, measured as part of the Maastricht Study, included age, sex, T2DM and education. Education was divided into low (no education, primary education and lower vocational education); medium (general secondary education, general vocational education and higher secondary and pre-university education) or high (higher vocational education and university). Presence of T2DM was measured by a glucose tolerance test and medication use^(18)^. Covariates used in the sensitivity analyses were housing value, household income, urbanicity and car ownership. Neighbourhood housing value and urbanicity were obtained from Statistics Netherlands. Participant housing value was calculated using the mean property value in Euro of the data collection period from 2010 to 2017. Household income was measured by self-reported net household income per month, and the equivalent income was computed taking account of the number of people in the household^(13,29)^. Urbanicity was divided into a 5-point scale: 1 (> 2500 addresses per km^2^), 2 (1500–2500), 3 (1000–1500), 4 (500–1000) and 5 (< 500). Car ownership was obtained from the Maastricht Study survey indicating yes or no.

### Statistical analyses

Descriptive characteristics of the total study population according to quartiles of the FEHI and DHD score were summarised as percentages to detect trends in demographics and socio-economic status. Separate linear regression analyses were used to assess the association between food outlet types, and the FEHI, with the DHD score. Model 1 was adjusted for age, sex and T2DM; model 2 was additionally adjusted for education level. Sub-components of the DHD and the FEHI were also analysed adjusting for age, sex, T2DM and education. Linear assumptions were met, and the residuals were found normally distributed after visual inspection of their histograms and P-P plots. Predicted values and residuals were plotted showing no indication of heteroscedasticity. The assumption of multicollinearity was examined by evaluating the correlation coefficients of the predictor variables and the variance inflation factors which showed no multicollinearity.

We performed several sensitivity analyses to verify the robustness of our findings taking account of variables that may alter participant geographic access and account for socio-economic indicators. First, we repeated the main analyses using 500 m and 1500 m buffer zones. Second, we ran the full model omitting T2DM as a covariate to determine if there was an overadjustment for this variable. Third, we substituted level of education for income and then average housing value by postcode separately. Fourth, we additionally adjusted for urbanicity which included age, sex and T2DM in the model. Finally, we ran the full model adjusting for vehicle ownership. Analyses were conducted using IBM SPSS Statistics for Windows, Version 27.0 (IBM Corp.). The threshold for significance was set at *P* < 0·05.

## Results

Table 1 displays participant data by FEHI score quartiles at the 1000 m buffer. Those within the lowest attained education group were predominately in the lowest quartile (32·6 %) compared to the healthiest quartile (13·8 %). The percentage of participants with T2DM was highest in the least healthy quartile (30·9 %) than the healthiest quartile (13·4 %). Table 2 presents the characteristics of the sample population categorised by DHD score dietary intake quartiles. In the total group, there was an equal number of male (50·1 %) and female participants with an average age of 59·8 years. Most participants with T2DM were both in the lowest DHD quartile (31·9 %) and in the lowest level of attained education (27·9 %).

**Table 1:** Participant characteristics by Food Environmental Healthiness Index (FEHI) quartiles

	Total population			FEHI quartiles											
				Quartile 1 (< –0·12)			Quartile 2 (–0·11–0·09)			Quartile 3 (–0·08–0·06)			Quartile 4 (> –0·05)		
				(Unhealthiest) *n* 2064			*n* 1640			*n* 2571			(Healthiest) *n* 1092		
	%	Mean		%	Mean		%	Mean		%	Mean		%	Mean	
Sex															
Male	50·10			28·80			22·40			33·70			15·00		
Female	49·90			27·40			22·40			36·10			14·20		
Age															
40–49	16·50			29·20			21·60			32·80			16·50		
50–59	31·50			27·60			20·30			36·20			16·00		
60–69	41·00			28·50			23·50			34·70			13·30		
70+	11·10			26·90			25·30			35·50			12·30		
Mean age		59·8	8·7		59·7	8·8		60·3	8·8		59·9	8·5		60·0	8·7
Education level															
Low	35·10			32·60			22·50			31·20			13·80		
Medium	27·70			29·50			21·10			34·30			15·10		
High	37·10			22·80			23·40			38·80			15·00		
Diabetes type II															
Yes	24·40			30·90			22·70			32·90			13·40		
No	75·60			27·20			22·20			35·60			15·00		

**Table 2:** Participant characteristics by Dutch Healthy Diet (DHD) score quartiles

				DHD score quartiles											
				Quartile 1 (< 73·5)			Quartile 2 (73·6–84·0)			Quartile 3 (84·0–94·5)			Quartile 4 (> 94·5)		
		Total population		(Unhealthiest) *n* 1842			*n* 1841			*n* 1842			(Healthiest) *n* 1842		
	%	Mean		%	Mean		%	Mean		%	Mean		%	Mean	
Sex															
Male	50·10			35·10			27·70			21·70			15·50		
Female	49·90			15·00			22·50			28·10			34·40		
Age															
40–49	16·50			33·20			25·20			22·30			19·30		
50–59	31·50			26·60			24·60			23·10			25·70		
60–69	41·00			21·20			25·00			26·60			27·20		
70+	11·10			22·30			26·50			27·80			23·50		
Mean age	59·8		8·7	64·2		7·5	60·0		8·7	60·7		8·4	60·6		8·2
Education level															
Low	34·80			27·90			25·70			25·50			20·90		
Medium	27·50			26·30			25·90			22·30			25·40		
High	37·70			21·20			23·80			26·30			28·70		
Diabetes type II															
Yes	24·40			31·90			27·50			23·80			16·90		
No	75·60			22·80			24·30			25·30			27·60		

Table 3 shows the association between food outlet type, FEHI and DHD score. There was a statistically significant positive association between density of restaurants and DHD score in model 1 adjusted for age and sex (B = 0·02; 95 % CI = 0·00, 0·04) but lost significance after further adjustment for level of education in model 2. There were no statistically significant associations between the other food outlet types nor the FEHI with the DHD score. There was no statistically significant association between the overall FEHI with the sub-components of the DHD (Table 4).

**Table 3:** Linear associations between the food environment and dietary intake

	Coefficient (B)	Model 1 95 % CI	*P*	Coefficient (B)	Model 2 95 % CI	*P*
Supermarkets	−0·09	−0·52, 0·34	0·647	−0·15	−0·58, 0·28)	0·680
Local food shops	−0·05	−0·21, 12	0·606	−0·10	−0·27, 0·06)	0·555
Restaurants	**0·02**	**0·00, 0·04**	0·043*	0·00	−0·02, 0·03)	0·547
Food delivery	0·03	−0·05, 0·11	0·360	0·02	−0·10, 0·06)	0·560
Convenience stores	0·00	−0·55, 0·54	0·949	−0·27	−0·82, 0·27)	0·324
Fast food	−0·02	−0·15, 0·12	0·816	−0·07	−0·20, 0·07)	0·321
FEHI	2·01	−1·17, 5·19	0·207	0·62	−2·54, 3·78)	0·702

FEHI, Food Environment Healthiness Index.

All exposure variables were analysed independently.

Model 1: adjusted for age, sex, and type II diabetes; Model 2: adjusted for model 1 + education.

*P < 0·05.

**Table 4:** Association between the Food Environment Healthiness Index and individual components of the Dutch Healthy Diet

	Coefficient	95 % CI	*P*
Fruit	−24·75	−55·69, 6·18	0·127
Vegetables	−19·04	−40·85, 2·77	0·097
Sugar-sweetened beverage	0·44	−29·44, 30·32	0·987
Processed meat	−3·02	−6·40, 0·36	0·080
Total Fat	0·69	−2·30, 3·69	0·651
Total fish	−0·36	−2·56, 1·85	0·753
Alcohol	1·19	−1·67, 4·05	0·414

Adjusted for age, sex, T2DM, education.

In the first sensitivity analyses, the model was repeated with environmental factors at the 500 m and 1500 m buffer (see online supplementary material, Supplemental Table 2). The only statistically significant associations with the DHD index were higher density of local food shops within 500 m was associated with a lower DHD index in model 1 (B = –0·09; CI = –0·18, 0·00); and a higher density of convenience stores was associated with a lower DHD index in model 2 (B = –0·33; CI = –0·66, –0·01). Within the 1500 m buffer, a higher density of restaurants was associated with a slightly higher DHD index in model 1 (B = 0·03; 95 % CI = 0·00, 0·06).

Second, analyses were performed with T2DM removed; and then, education was substituted for income; and lastly, education was substituted with housing value which did not materially change the results (see online supplementary material, Supplemental Table 3–5). We additionally adjusted for urbanicity (see online supplementary material, Supplemental Table 6) in which we did not find significant associations. Further adjusting for car ownership, we found a positive association with restaurants (B = 0·03; 95 % CI = 0·01, 0·05) but not with the other food outlets or overall FEHI (see online supplementary material, Supplemental Table 7).

## Discussion

In this study, we evaluated the associations between the neighbourhood food environment and participant diet quality (DHD score). A comprehensive measure of the food environment integrating distance, density and healthiness of food outlets did not result in a clear association between the food environment and diet quality. There were few statistically significant relationships with rather small coefficients between food outlet type and diet quality, but one ought to account for the multiple tests that we performed. In addition, there was no consistent trend in the direction of these associations. Using two additional buffer sizes and looking at separate outlets did not reveal a particular pattern either. In evaluating the sub-components of the DHD, there was no association between the neighbourhood FEHI on these specific food items (e.g. fruits, vegetables, sugar-sweetened beverages). We also adjusted for socio-economic variables that included income level, housing value, urbanicity and car ownership which further bolstered our findings as they did not yield significant relationships with participant diet.

Contrary to our hypothesis, a healthier food environment in our study was not associated with a healthier diet as defined by a higher DHD score. Particularly, we expected to find healthier diet quality in those living within close access to supermarkets. Though supermarkets generally contain far more produce than any type of food outlet, there was no finding that living in proximity to these retailers coincided with healthier diet quality. The FEHI assigns these outlets the fourth highest score though it has been documented in previous studies that large grocery stores have mixed results with nearby residents in terms of diet and obesity outcomes^(30–32)^. One explanation that Hallum and colleagues proposed was that having access to a high-quality supermarket does not guarantee that a consumer will not pursue cheaper, less healthy options at these stores when there are financial constraints^(33)^. Another factor is that these markets often contain as much as, if not more ultra-processed foods and confectionary items than shops that are categorised as unhealthy. A final consideration is that a substantial number of individuals will travel to their preferred market or restaurant well beyond their neighbourhood^(34–38)^. However, in the current study, we found that adjusting for vehicle ownership was not associated with the overall food environment. Future work should consider evaluating individual utilisation within their food environment. On the other spectrum of food quality, we did not find that areas with a higher density of unhealthy food outlets, particularly fast food, had a significant association with worse diet quality. This relationship was also observed by two recent studies which were sampled in the Netherlands^(39,40)^.

**Figure 2:**
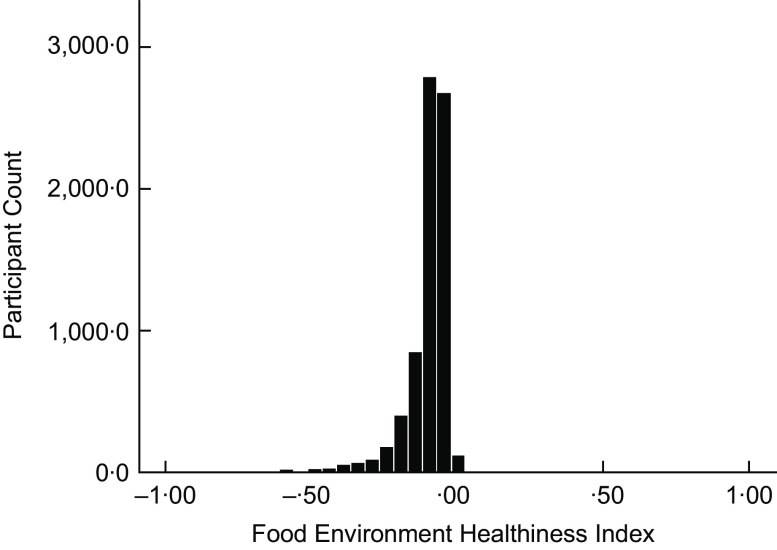
Mean distribution of the neighbourhood food environment by participants using the FEHI (unhealthy to unhealthiest: −0.1 to −5.0, healthy to healthiest: 0.1 to 5.0). X-axis was truncated as there were no values beyond −1.0 or 1.0+. FEHI, Food Environment Healthiness Index.

The geographical layout of our study region displayed small areas that are unhealthy but within a narrow range overall in the city centre and throughout the greater Maastricht area with little variation in the food environment (Figs 1 and 2). We postulate that despite these small pockets that feature higher densities of less healthy food, the participants still had reasonable access to healthier food outlets in their immediate vicinity. Though this was not a priority in our study, it appears we did not find a region that spanned more than several neighbourhood blocks with exceedingly unhealthy food outlet exposure. The majority of our study sample region was slightly negative according to the FEHI scale suggesting a marginally unhealthy food environment (Fig. 2). Thus, given the thorough collection of food retailers, it appears we did not find what would suggest the presence of a food desert.

Previous studies by and large tend to contrast adjacent neighbourhood areas that vastly differ on the socio-economic gradient resulting in consistent disparity in dietary consumption and resulting obesity outcomes^(10,41,42)^. The geographical layout of our study region also showed a diverse variation in socio-economic status by neighbourhood postal code. That variation was sufficient to allow a strong association between the average housing value and the odds of T2DM^(43)^. Furthermore, in the current study, we found that participants with lower attained education lived in relatively unhealthier food environments. Still, the variation in the food environment and the covariation with area socio-economic circumstances in the Netherlands might be diminutive in comparison with the USA. Other literature suggests that socio-economic and racial disparities in European societies have a lesser effect on food access than in North America^(44)^. In addition, unequal access to a variety of food retailers is less common in Europe, even in neighbourhoods with a high number of minorities^(14)^. The distribution of participants on the FEHI shows that they live in a marginally unhealthy food environment (Fig. 2). In regions with a high density of unhealthy food, we believe they still have access to a healthier option for nearly every unhealthy outlet within their neighbourhoods. Future work that better accounts for societal differences will be beneficial, particularly when setting out to determine the full potential of food environments in influencing individuals towards unhealthy dietary intakes.

There were several limitations to our study. First, this was a cross-sectional design and we were unable to infer causality between the food environment and dietary intake. Second, self-reported diet is well-known to be susceptible to inaccuracy and social desirability bias among others. However, given the exhaustive cross-disciplinary battery of questionnaires and medical exams the participants underwent, this was the most feasible and efficient method of gathering dietary intake information from each participant. The validated FFQ where the DHD score was obtained from was comprehensive, consisting of 253 food items. Third, we analysed the six most common food outlet types patronised by the participants. This may have omitted specialty markets and specific shops that our participants may frequent. The additional analysis of the FEHI was inclusive of all food retailers in the study region and thus, all assortments of food markets in our study area were represented in the analysis. Fourth, we quantified residential exposure to the food environment, whereas this may not have covered respondents’ actual activity space. We were not able to separately explore the workplace food environment. Given that half of our study population still have paid jobs, the workplace food environment might additionally have affected participant dietary quality. Finally, the FEHI assigned a score to a food outlet type by what it assumes it offers. Scales like these are unable to account for individual retailer variation within each category especially if it is not a recognised chain store. Certain establishments may be rated too low or high as a result. Thus, measures like these are not without imperfections and need to be continuously refined for greater validity.

The strength of this study was the large sample size which provided adequate power. We were able to develop a unique radius of food shops as well as calculate the food environment based on an individual’s home address. This advantage was a more precise, geographical representation as opposed to assigning the participant to a postal code or neighbourhood tract where their location may possibly overlap several defined geographic areas within their home radius. Another strength was that the directory of food establishments was obtained from the LOCATUS database which is regularly updated every 2–3 years on average and showed good to excellent agreement statistics (> 0·71) with field audit data for all three level of analysis (i.e. location, classification and both combined) and across urban as well as rural areas^(45)^. This comprehensive listing allowed us to account for food retailers that may have closed, moved or recently opened. This study was one of few instances where the complete food environment was observed as opposed to specific retailer types. We were also able to differentiate rural *v*. urban residents accounting for individuals who may find it more or less necessary to drive longer distances to seek common items. Finally, we adjusted for common socio-economic variables that may alter purchasing and dietary consumption including housing value, income and education.

## Conclusion

The food environment in the Maastricht area appeared marginally unhealthy, but the differences in the food environment were not related to the quality of food that participants reported as intake. Before rejecting the food environment hypothesis, research is needed with better measures in areas where there is more variation in the food environment, as in the case in the USA. Research on the food environment should further evaluate longitudinal changes, utilise larger spatial variance in food outlet exposures, perform complete participant dietary measures and continue to account for all food outlet types to investigate a causal explanation.

## Supplementary material

For supplementary material accompanying this paper visit https://doi.org/10.1017/S1368980023001180. click here to view supplementary material

## References

[1] Kaptoge S , Pennells L , de Bacquer D et al. (2019) World Health Organization cardiovascular disease risk charts: revised models to estimate risk in 21 global regions. Lancet Global Health 7, e1332–1345. 31488387 10.1016/S2214-109X(19)30318-3 PMC7025029

[2] Caspi CE , Sorensen G , Subramanian SV et al. (2012) The local food environment and diet: a systematic review. Health Place 18, 1172–1187. 22717379 10.1016/j.healthplace.2012.05.006 PMC3684395

[3] Drewnowski A , Arterburn D , Zane J et al. (2019) The Moving to Health (M2H) approach to natural experiment research: a paradigm shift for studies on built environment and health. SSM – Popul Health 7, 100345. 30656207 10.1016/j.ssmph.2018.100345 PMC6329830

[4] Vadiveloo MK , Sotos-Prieto M , Parker HW et al. (2021) Contributions of food environments to dietary quality and cardiovascular disease risk. Curr Atheroscler Rep 17, 4. 10.1007/s11883-021-00912-9 33594516

[5] Bodor JN , Rose D , Farley TA et al. (2008) Neighbourhood fruit and vegetable availability and consumption: the role of small food stores in an urban environment. Public Health Nutr 11, 413–420. 17617930 10.1017/S1368980007000493

[6] Moore LV , Diez Roux AV , Nettleton JA et al. (2008) Associations of the local food environment with diet quality--a comparison of assessments based on surveys and geographic information systems: the multi-ethnic study of atherosclerosis. Am J Epidemiol 167, 917–924. 18304960 10.1093/aje/kwm394 PMC2587217

[7] Morland K , Diez Roux AV & Wing S (2006) Supermarkets, other food stores, and obesity: the atherosclerosis risk in communities study. Am J Prev Med 30, 333–339. 16530621 10.1016/j.amepre.2005.11.003

[8] Jiao J , Moudon AV , Kim SY et al. (2015) Health implications of adults’ eating at and living near fast food or quick service restaurants. Nutr Diabetes 5, e171–e176. 26192449 10.1038/nutd.2015.18 PMC4521173

[9] Spence JC , Cutumisu N , Edwards J et al. (2009) Relation between local food environments and obesity among adults. BMC Public Health 9, 192. 19538709 10.1186/1471-2458-9-192 PMC2708156

[10] Beaulac J , Kristjansson E & Cummins S (2009) A systematic review of food deserts, 1966–2007. Prev Chronic Dis 6, A105. 19527577 PMC2722409

[11] Larson NI , Story MT & Nelson MC (2009) Neighborhood environments: disparities in access to healthy foods in the U.S. Am J Preventative Med 36, 74–81. 10.1016/j.amepre.2008.09.025 18977112

[12] Moore LV & Diez Roux AV (2006) Associations of neighborhood characteristics with the location and type of food stores. Am J Public Health 96, 325–331. 16380567 10.2105/AJPH.2004.058040 PMC1470485

[13] OECD (2021) Inequality – Income Inequality – OECD Data. https://data.oecd.org/inequality/income-inequality.htm (accessed June 2021).

[14] Helbich M , Schadenberg B , Hagenauer J et al. (2017) Food deserts? Healthy food access in Amsterdam. Appl Geogr 83, 1–12.

[15] Regan G , Lee RE , Booth K et al. (2006) Obesogenic influences in public housing: a mixed-method analysis. Am J Health Promot 20, 282–290. 16555802 10.4278/0890-1171-20.4.282

[16] Walker RE , Keane CR & Burke JG (2010) Disparities and access to healthy food in the United States: a review of food deserts literature. Health Place 16, 876–884. 20462784 10.1016/j.healthplace.2010.04.013

[17] Giskes K , van Lenthe F , Avendano-Pabon M et al. (2010) A systematic review of environmental factors and obesogenic dietary intakes among adults: are we getting closer to understanding obesogenic environments? Obes Rev 12, e95–106. 10.1111/j.1467-789X.2010.00769.x 20604870

[18] Schram MT , Sep SJS , van der Kallen CJ et al. (2014) The Maastricht Study: an extensive phenotyping study on determinants of type 2 diabetes, its complications and its comorbidities. Eur J Epidemiol 29, 439–451. 24756374 10.1007/s10654-014-9889-0

[19] van Dongen MC , Wijckmans-Duysens NEG , den Biggelaar LJ et al. (2019) The Maastricht FFQ: development and validation of a comprehensive food frequency questionnaire for the Maastricht study. Nutrition 62, 39–46. 30826598 10.1016/j.nut.2018.10.015

[20] Looman M , Feskens EJM , de Rijk M et al. (2017) Development and evaluation of the Dutch Healthy Diet index 2015. Public Health Nutr 20, 2289–2299. 28625202 10.1017/S136898001700091X PMC10261559

[21] Lakerveld J , Wagtendonk A , Vaartjes I et al. (2020) Deep phenotyping meets big data: the Geoscience and Health Cohort COnsortium (GECCO) data to enable exposome studies in The Netherlands. Int J Health Geographics 19, 1. 10.1186/s12942-020-00235-z PMC7662022 33187515

[22] Locatus (2015) Retail Facts 2015. https://locatus.com/en/applications/retail-outlet-explorer/ (accessed March 2021).

[23] Locatus (2017) Retail Facts 2017. https://locatus.com/en/applications/retail-outlet-explorer/ (accessed March 2021).

[24] Locatus (2010) Retail Facts 2010. https://locatus.com/en/applications/retail-outlet-explorer/ (accessed March 2021).

[25] Locatus (2012) Retail Facts 2012. https://locatus.com/en/applications/retail-outlet-explorer/ (accessed March 2021).

[26] Poelman M (2018) Food Environment ‘Healthiness Scores’ for The Netherlands. White Paper.

[27] James P , Berrigan D , Hart JE et al. (2014) Effects of buffer size and shape on associations between the built environment and energy balance. Health Place 27, 162–170. 24607875 10.1016/j.healthplace.2014.02.003 PMC4028172

[28] Pinho MGM , Mackenbach JD , Charreire H et al. (2019) Comparing different residential neighborhood definitions and the association between density of restaurants and home cooking among Dutch adults. Nutrients 8, 1796. 10.3390/nu11081796 PMC6722945 31382624

[29] Qi Y , Koster A , van Boxtel M et al. (2019) Adulthood socioeconomic position and type 2 diabetes mellitus-A comparison of education, occupation, income, and material deprivation: the Maastricht study. Int J Environ Res Public Health 16, 1435–1447. 31018480 10.3390/ijerph16081435 PMC6517950

[30] Gase LN , DeFosset AR , Smith LV et al. (2014) The association between self-reported grocery store access, fruit and vegetable intake, sugar-sweetened beverage consumption, and obesity in a racially diverse, low-income population. Front Public Health 2, 229. 25426485 10.3389/fpubh.2014.00229 PMC4227465

[31] Hattori A , An R & Sturm R (2013) Neighborhood food outlets, diet, and obesity among California adults, 2007 and 2009. Prev Chronic Dis 10, E35. 23489640 10.5888/pcd10.120123 PMC3600873

[32] Caspi CE , Lenk K , Pelletier JE et al. (2017) Association between store food environment and customer purchases in small grocery stores, gas-marts, pharmacies and dollar stores. Int J Behav Nutr Phys Act 14, 75. 28583131 10.1186/s12966-017-0531-x PMC5460502

[33] Hallum SH , Hughey SM , Wende ME et al. (2020) Healthy and unhealthy food environments are linked with neighbourhood socio-economic disadvantage: an innovative geospatial approach to understanding food access inequities. Public Health Nutr 23, 3190–3196. 32782060 10.1017/S1368980020002104 PMC10200448

[34] Aggarwal A , Cook AJ , Jiao J et al. (2014) Access to supermarkets and fruit and vegetable consumption. Am J Public Health 104, 917–923. 24625173 10.2105/AJPH.2013.301763 PMC3987578

[35] Cannuscio CC , Tappe K , Hillier A et al. (2013) Urban food environments and residents’ shopping behaviors. Am J Prev Med 45, 606–614. 24139774 10.1016/j.amepre.2013.06.021

[36] Gustafson A , Christian JW , Lewis S et al. (2013) Food venue choice, consumer food environment, but not food venue availability within daily travel patterns are associated with dietary intake among adults, Lexington Kentucky 2011. Nutr J 12, 17. 23360547 10.1186/1475-2891-12-17 PMC3571876

[37] Moran AJ , Gu Y , Clynes S et al. (2020) Associations between governmental policies to improve the nutritional quality of supermarket purchases and individual, retailer, and community health outcomes: an integrative review. Int J Environ Res Public Health 17, 20. 10.3390/ijerph17207493 PMC7602424 33076280

[38] Sohi I , Bell BA , Liu J et al. (2014) Differences in food environment perceptions and spatial attributes of food shopping between residents of low and high food access areas. J Nutr Educ Behav 46, 241–249. 24560861 10.1016/j.jneb.2013.12.006 PMC4205937

[39] Harbers MC , Beulens JWJ , Boer JM et al. (2021) Residential exposure to fast-food restaurants and its association with diet quality, overweight and obesity in the Netherlands: a cross-sectional analysis in the EPIC-NL cohort. Nutr J 20, 56. 34134701 10.1186/s12937-021-00713-5 PMC8210363

[40] van Rongen S , Poelman MP , Thornton L et al. (2020) Neighbourhood fast food exposure and consumption: the mediating role of neighbourhood social norms. Int J Behav Nutr Phys Act 17, 61. 32404102 10.1186/s12966-020-00969-w PMC7218623

[41] Zachary DA , Palmer AM , Beckham SW et al. (2013) A framework for understanding grocery purchasing in a low-income urban environment. Qual Health Res 23, 665–678. 23443333 10.1177/1049732313479451

[42] Zenk SN , Odoms-Young AM , Dallas C et al. (2011) ‘You have to hunt for the fruits, the vegetables’: environmental barriers and adaptive strategies to acquire food in a low-income African American neighborhood. Health Educ Behav 38, 282–292. 21511955 10.1177/1090198110372877 PMC3709968

[43] Consolazio D , Koster A , Sarti S et al. (2020) Neighbourhood property value and type 2 diabetes mellitus in the Maastricht study: a multilevel study. PLoS ONE 15, 6. 10.1371/journal.pone.0234324 PMC7279598 32511267

[44] Cummins S & Macintyre S (2006) Food environments and obesity – neighbourhood or nation? Int J Epidemiol 35, 100–104. 16338945 10.1093/ije/dyi276

[45] Canalia C , Pinho M , Lakerveld J et al. (2020) Field validation of commercially available food retailer data in the Netherlands. Int J Environ Res Public Health 17, 1946. 32188152 10.3390/ijerph17061946 PMC7143735

