# Supplementary material for: The association between the food environment and adherence to healthy diet quality: the Maastricht Study
Source: Public Health Nutr. 2023 Jun 21;26(9):1775–83. doi: 10.1017/S1368980023001180 (PMC10478064; doi:10.1017/S1368980023001180)
Supplement: Supplementary file 1 [file S1368980023001180sup001.docx]

**Supplementary Table 1:** Food Environmental Healthiness Score assignments

| **Food outlet** | **Scores** |
| --- | --- |
| Fruit/Vegetable farmstand | 4.8 |
| Fish Market | 2.8 |
| Nutbar | 2.2 |
| Supermarket | 1.8 |
| Traditional Oriental Market | 1.5 |
| Coffee shop | 1.3 |
| Baker | 1.2 |
| Health Food store | 1.0 |
| Poultry Shop | 0.6 |
| Delicatessen | 0.2 |
| Butcher Shop | -0.4 |
| Hotel-restaurant | -0.9 |
| Restaurant | -0.9 |
| Cheese shop | -1.3 |
| Lunchroom/ Coffeebar (Bijvoorbeeld: Bagels & Beans, Délifrance, Panache, Subway, Bakkerswinkel, Doppio, Starbucks, Coffee company) | -1.5 |
| Café-restaurant | -1.9 |
| Café | -2.3 |
| Pancake-restaurant | -3.2 |
| Takeout/Delivery (ex. Chinese, Thai, pizza/pasta, Taco’s) | -3.7 |
| Ice cream parlour | -3.9 |
| Chocolate store (e.g. bonbonnière, Leonidas) | -4.3 |
| Pieshop | -4.4 |
| Tobacco and Literature shop | -4.5 |
| Gas Station | -4.5 |
| Liquor Store | -4.6 |
| Confectionary | -4.7 |
| Grillroom/Shawarma | -4.8 |
| Snackbar / fast-food outlets | -4.9 |
| Minisupermarkets includes:  Turkish supermarket  Neighborhood supermarket  Nightshop  Remaining stores | 2.0  0.3  -2.1  0.1 |

| Supplementary Table 2: Linear associations between the food environment and dietary intake at 500m and 1500m buffers | | | | | | |
| --- | --- | --- | --- | --- | --- | --- |
|  | Coefficient | **Model 1**  95% CI | P Value | Coefficient | **Model 2**  95% CI | P Value |
| Supermarkets |  |  |  |  |  |  |
| 500m | -.17 | (-.36, .01) | .070 | -.15 | (.-34, .04) | .116 |
| 1500m | -.07 | (-.63, .48) | .800 | -.28 | (-.83, .28) | .332 |
| Local Food |  |  |  |  |  |  |
| 500m | **-.09** | **(-.18, -.00)** | **.047*** | -.09 | (-.18, .00) | .052 |
| 1500m | -.04 | (-.25, .17) | .686 | -.15 | (-.36, .06) | .166 |
| Restaurants |  |  |  |  |  |  |
| 500m | .01 | (-.00, .03) | .092 | .01 | (-.01, .02) | .552 |
| 1500m | **.03** | **(.00, .06)** | **.031*** | .01 | (-.02, .04) | .642 |
| Food Delivery |  |  |  |  |  |  |
| 500m | -.03 | (-.24, .18) | .786 | -.13 | (.34, .08) | .230 |
| 1500m | .02 | (-.03, .07) | .454 | -.02 | (-.07, .03) | .404 |
| Convenience Stores |  |  |  |  |  |  |
| 500m | -.29 | (-.62, .04) | .082 | **-.33** | **(-.66, -.01)** | **.046*** |
| 1500m | .08 | (-.66, .81) | .839 | -.36 | (-1.09, .38) | .338 |
| Fast Food |  |  |  |  |  |  |
| 500m | -.09 | (-.17, .00) | .058 | -.08 | (-.17, .00) | .058 |
| 1500m | -.04 | (-.25, .17) | .686 | -.07 | (-.25, -.10) | .410 |
| FEHI |  |  |  |  |  |  |
| 500m | 1.70 | (-.11, 3.50) | .066 | .95 | (-.85, 2.75) | .300 |
| 1500m | 3.48 | (-1.42, 8.39) | .164 | 1.57 | (-3.30, 6.44) | .527 |
| **Model 1:** Adjusted for age, sex, T2DM; **Model 2:** Model 1 + education. **FEHI:** Food Environment Healthiness Index  **P*<0•05 | | | | | | |

| Supplementary Table 3: Linear associations between the food environment and dietary intake at 1000m buffer (Without Type 2 Diabetes adjustment) | | | |
| --- | --- | --- | --- |
|  | Coefficient | 95% CI | P-Value |
| Supermarkets | -.16 | (-.59, .27) | .459 |
| Local Food Stores | -.10 | (-.27, .06) | .227 |
| Restaurants | .01 | (-.01, .03) | .481 |
| Food Delivery | -.02 | (-.10, .07) | .714 |
| Convenience Stores | -.23 | (-.77, .32) | .411 |
| Fast Food | -.07 | (-.20, .07) | .339 |
| FEHI | .64 | (-2.53, 3.81) | .691 |
| Adjusted for age, sex, education | | | |
| **P*<0•05 | | | |

| Supplementary Table 4: Linear Associations between the food environment and dietary intake adjusted for income level at 1000m buffer | | | |
| --- | --- | --- | --- |
|  | Coefficient | 95% CI | P-Value |
| Supermarkets | .33 | (-.16, .82) | .182 |
| Local Food Stores | .02 | (-.17, .21) | .851 |
| Restaurants | .02 | (-.01, .04) | .144 |
| Food Delivery | .00 | (-.09, .10) | .949 |
| Convenience Stores | .18 | (-.44, .80) | .569 |
| Fast Food | .05 | (-.10, .21) | .498 |
| FEHI | -.09 | (-3.80, 3.62) | .961 |
| Adjusted for age, sex, T2DM, income | | | |
| **P*<0•05 | | | |

| Supplementary Table 5: Linear associations between the food environment and dietary intake adjusted for housing value at 1000m buffer | | | |
| --- | --- | --- | --- |
|  | Coefficient | 95% CI | P-Value |
| Supermarkets | .10 | (-.34, .55) | .643 |
| Local Food Stores | -.06 | (-.22, .11) | .492 |
| Restaurants | .02 | (-.01, .04) | .171 |
| Food Delivery | .01 | (-.07, .09) | .776 |
| Convenience Stores | -.00 | (-.54, .54) | .997 |
| Fast Food | -.00 | (-.14, .14) | .989 |
| FEHI | 2.56 | (-.85, 5.98) | .141 |
| Adjusted for age, sex, T2DM, housing value | | | |
| **P*<0•05 | | | |

| Supplementary Table 6: Linear associations between the food environment and dietary intake adjusted for urbanicity at 1000m buffer | | | |
| --- | --- | --- | --- |
|  | Coefficient | 95% CI | P-Value |
| Supermarkets | .34 | (-.21, .88) | .227 |
| Local Food Stores | .03 | (-.17, .23) | .769 |
| Restaurants | .02 | (-.01, .04) | .115 |
| Food Delivery | .05 | (-.05, .14) | .351 |
| Convenience Stores | .13 | (-.50, .76) | .687 |
| Fast Food | .05 | (-.12, .21) | .547 |
| FEHI | 1.45 | (-1.77, 4.66) | .378 |
| Adjusted for age, sex, T2DM, education, urbanicity | | | |
| **P*<0•05 | | | |

| Supplementary Table 7: Linear associations between the food environment and dietary intake adjusted for vehicle ownership at 1000m buffer | | | |
| --- | --- | --- | --- |
|  | Coefficient | 95% CI | P-Value |
| Supermarkets | .11 | (-.34, .56) | .629 |
| Local Food Stores | .01 | (-.17, .18) | .945 |
| Restaurants | **.03** | **(.01, .05)** | **.012*** |
| Food Delivery | .05 | (-.04, .13) | .290 |
| Convenience Stores | .15 | (-.42, .72) | .600 |
| Fast Food | .04 | (-.10, .18) | .577 |
| FEHI | 2.34 | (-1.01, 5.69) | .172 |
| Adjusted for age, sex, T2DM, education, vehicle ownership | | | |
| **P*<0•05 | | | |
